# Integrative analysis of pathogen detection, antimicrobial resistance, virulence, and host response in severe infections using metagenomic next-generation sequencing

**DOI:** 10.3389/fcimb.2026.1786413

**Published:** 2026-04-10

**Authors:** Xu-Guang Chen, Lei Zhou, Kai Duan, Sheng-Yi Shi, Abudurexiti Subi, Han-Wen Sun, Yi-Ming Lu, Lan Hu, Zhi-Tao Yang

**Affiliations:** 1 Emergency Department of Shanghai Ruijin Hospital, Shanghai Jiaotong University School of Medicine, Shanghai, China; 2 Emergency Department of Yueyang Central Hospital, Hunan, China

**Keywords:** antimicrobial resistance, infections in ICU patients, host immune response, metagenomic next-generation sequencing (mNGS), precision medicine, virulence factor

## Abstract

**Background:**

Metagenomic next-generation sequencing (mNGS) offers unbiased pathogen detection. However, its integrative value in simultaneously revealing resistance, virulence, and host-response interplay in Intensive Care Unit(ICU)-infected patients remains underexplored.

**Methods:**

In this retrospective cohort study of 156 ICU-infected patients, we compared the diagnostic performance of mNGS against conventional microbiological testing (CMT). We analyzed mNGS-derived antibiotic resistance genes (ARGs) and virulence factors (VFs) and correlated them with host immune-inflammatory markers and clinical outcomes.

**Results:**

mNGS demonstrated a significantly higher positive detection rate (89.7% vs. 67.3%, P < 0.001) and clinical concordance (75.6% vs. 35.9%, P < 0.001) than CMT. It revealed a high mixed-infection rate (72.1%). ARGs were detected in 49.0% of bacterial infections, predominantly β-lactamase genes, showing 72.0% concordance with phenotypic susceptibility. Key VFs (e.g., *rmpA* in *K. pneumoniae*) were identified. Based on mNGS results, 47.4% of patients had their antimicrobial therapy adjusted.

**Conclusion:**

mNGS provides a comprehensive diagnostic tool by integrating pathogen identification, resistance and virulence profiling, and host-response context, enabling more precise and timely management of ICU-infected patients.

## 1 Introduction

Infectious diseases remain one of the leading causes of morbidity and mortality worldwide. Especially for critically ill and immunocompromised patients, rapid and accurate diagnosis of infection is key to successful treatment (Singer et al., 2016). Conventional microbiological methods (e.g., culture, serology, targeted PCR) have limitations such as long turnaround times, limited pathogen coverage, and susceptibility to prior antibiotic treatment, making it difficult to meet urgent clinical needs (Zeng et al., 2024). This diagnostic dilemma often leads to the overuse of broad-spectrum antibiotics, exacerbating the global epidemic of bacterial resistance (Ferreyra et al., 2026).

Metagenomic next-generation sequencing (mNGS) technology does not require predefined primers and can perform high-throughput sequencing of all nucleic acid sequences in clinical samples. In theory, it can detect all potential pathogens (including bacteria, viruses, fungi, parasites, and atypical pathogens) in an unbiased manner, and has become a powerful tool in recent years for addressing complex, difficult, and critical infections (Wilson et al., 2019; Gu et al., 2021; Elbehiry and Abalkhail, 2025). Numerous studies have confirmed that mNGS is significantly superior to conventional methods in pathogen detection rate, particularly demonstrating great advantages in diagnosing mixed infections, rare pathogen infections, and culture-negative infections (Han et al., 2019; Flurin et al., 2022). However, the value of mNGS extends far beyond pathogen detection; the massive data it generates contains valuable information about pathogen resistance genes (ARGs) and virulence factors (VFs) (Charalampous et al., 2019; Diao et al., 2022). These molecular characteristics are closely related to the occurrence, development, and outcome of infections. For example, the presence of carbapenemase genes (e.g., *bla*KPC, *bla*NDM) predicts potential failure of carbapenem antibiotic therapy (Reyes et al., 2023), while virulence factors specific to hypervirulent *Klebsiella pneumoniae* (hvKP) (e.g., *rmpA/A2*, *iucABCD-iutA*) are closely associated with the invasive syndromes and sepsis it causes (Xu et al., 2024). Simultaneously, the host’s immune status, such as lymphocyte depletion, is not only a breeding ground for opportunistic infections but may also influence pathogen evolutionary pressure, promoting the selection of resistant or hypervirulent strains (Dickson and Huffnagle, 2015).

Currently, research integrating the pathogen, resistance, and virulence information provided by mNGS with host immune-inflammatory indicators is still relatively scarce. This study aims to systematically evaluate the comprehensive diagnostic value of mNGS, deeply explore the association between pathogen molecular characteristics and host response, and assess its practical significance in guiding clinical precision treatment, by analyzing the clinical and mNGS data of 156 patients with infectious diseases.

## 2 Materials and methods

### 2.1 Study subjects

Briefly, during the study period (May 2023 - May 2025), a total of 412 patients in the ICU were initially assessed for suspected serious infections and mNGS testing. After applying the inclusion and exclusion criteria, 156 patients were ultimately included in the final analysis(See **Supplementary Figure 1** for the detailed patient selection flow diagram).Inclusion criteria: (1) Age ≥18 years; (2) Meeting clinical diagnostic criteria for infectious diseases (e.g., fever, elevated inflammatory markers, imaging suggestive of infection, etc.); (3) Concurrent mNGS and conventional microbiological testing (including bacterial/fungal culture, serology, targeted PCR, etc.) were performed. Exclusion criteria: (1) Incomplete clinical data; (2) Final diagnosis of non-infectious disease. This study was reviewed and approved by the Ethics Committee of Ruijin Hospital Affiliated to Shanghai Jiao Tong University School of Medicine (Approval No.: (2025) Lin Lun Shen Di (591) Hao), with patient informed consent waived.

### 2.2 Research methods

#### 2.2.1 Conventional microbiological testing

All samples underwent bacterial/fungal culture, smear microscopy, and antimicrobial susceptibility testing (using the VITEK 2 Compact system or disk diffusion method) according to standard operating procedures. Serological and targeted PCR testing were performed based on clinical needs.

#### 2.2.2 Metagenomic next-generation sequencing and bioinformatics analysis

Patient samples such as bronchoalveolar lavage fluid (BALF), blood, cerebrospinal fluid (CSF), or others were collected. Total nucleic acid was extracted using a DNA/RNA co-extraction kit. DNA and RNA libraries were constructed, followed by high-throughput sequencing on the Illumina NextSeq 550 platform. After quality control, the sequencing data were aligned to the human reference genome (HG19) to remove human sequences. The remaining non-human sequences were then aligned to specialized microbial databases (NCBI NT/NR databases) for species identification and abundance analysis. Simultaneously, resistance gene databases (CARD) and virulence factor databases (VFDB) were used to annotate the sequences and analyze the presence of resistance genes and virulence factors.(For details, please refer to **Supplementary File 1**).

#### 2.2.3 Clinical data collection

Patient baseline data (age, sex, underlying diseases), clinical symptoms, laboratory results (blood routine, inflammatory markers, lymphocyte subsets, etc.), imaging data, treatment course, and clinical outcomes were collected.

#### 2.2.4 Clinical adjudication

Two independent senior infectious disease physicians adjudicated the final etiological diagnosis based on all available clinical data, including microbiology, imaging, treatment response, and follow-up. Discrepancies were resolved by consensus with a third reviewer. Reviewers were blinded to mNGS resistance and virulence annotation results.

### 2.3 Statistical analysis

SPSS 26.0 and Python 3.13.5 software were used for statistical analysis. Normally distributed measurement data are expressed as mean ± standard deviation (mean ± SD), with intergroup comparisons using the t-test; non-normally distributed measurement data are expressed as median (interquartile range) [M (Q1, Q3)], with intergroup comparisons using the Mann-Whitney U test. Count data are expressed as number (percentage) [n (%)], with intergroup comparisons using the χ² test or Fisher’s exact test. The Kappa test was used to analyze the consistency between mNGS and culture results. A P-value < 0.05 was considered statistically significant.

## 3 Results

### 3.1 Patient baseline characteristics, sample types, and immune-inflammatory status

The 156 ICU-infected patients finally included in this study were overall critically ill. Their basic characteristics are detailed in **Table 1**. The average age of patients was 65.5 ± 13.9 years, predominantly male (102 cases, 65.4%), reflecting that elderly males are a high-risk group for ICU-infected patients. Patients had complex and diverse underlying diseases, with the top four being hypertension (83 cases, 53.2%), diabetes (36 cases, 23.1%), neurological diseases (22 cases, 14.1%), and autoimmune diseases (22 cases, 14.1%). These comorbidities form the pathological basis for susceptibility to infection and progression to critical illness.

**Table 1:** Baseline characteristics of the 156 ICU-infected patients.

Characteristic	Value
Demographics	
Age, years, mean ± SD	65.5 ± 13.9
Male, n (%)	102 (65.4)
Primary Clinical Diagnosis, n (%)	
Severe Pneumonia	97 (62.2)
Bloodstream infection	19 (12.2)
Intracranial Infection	17 (10.9)
Other Infections	23 (14.7)
Disease Severity & Support	
APACHE II score, mean ± SD	24.6 ± 6.4
SOFA score, mean ± SD	13.5± 3.4
Septic Shock, n (%)	79 (50.6)
Mechanical Ventilation, n (%)	115 (73.7)
Duration of Mechanical Ventilation, days, median (IQR)	17 (6, 51)
ICU Length of Stay, days, median (IQR)	17 (10, 40)
Key Comorbidities, n (%)	
Hypertension	83 (53.2)
Diabetes Mellitus	36 (23.1)
Chronic lung disease	18 (11.5)
Malignant neoplasm	16 (10.3)
Chronic liver disease	6 (3.8)
Autoimmune disease	22(14.1)
Neurological disorders	22(14.1)
Clinical Outcome, n (%)	
Improved	104 (66.7)
Deceased	52 (33.3)

APACHE II, Acute Physiologic Score and Chronic Health Evaluation score; SOFA, Sequential Organ Failure Assessment score.

### 3.2 Pathogen spectrum and mixed infection analysis based on mNGS

**Figure 1:**
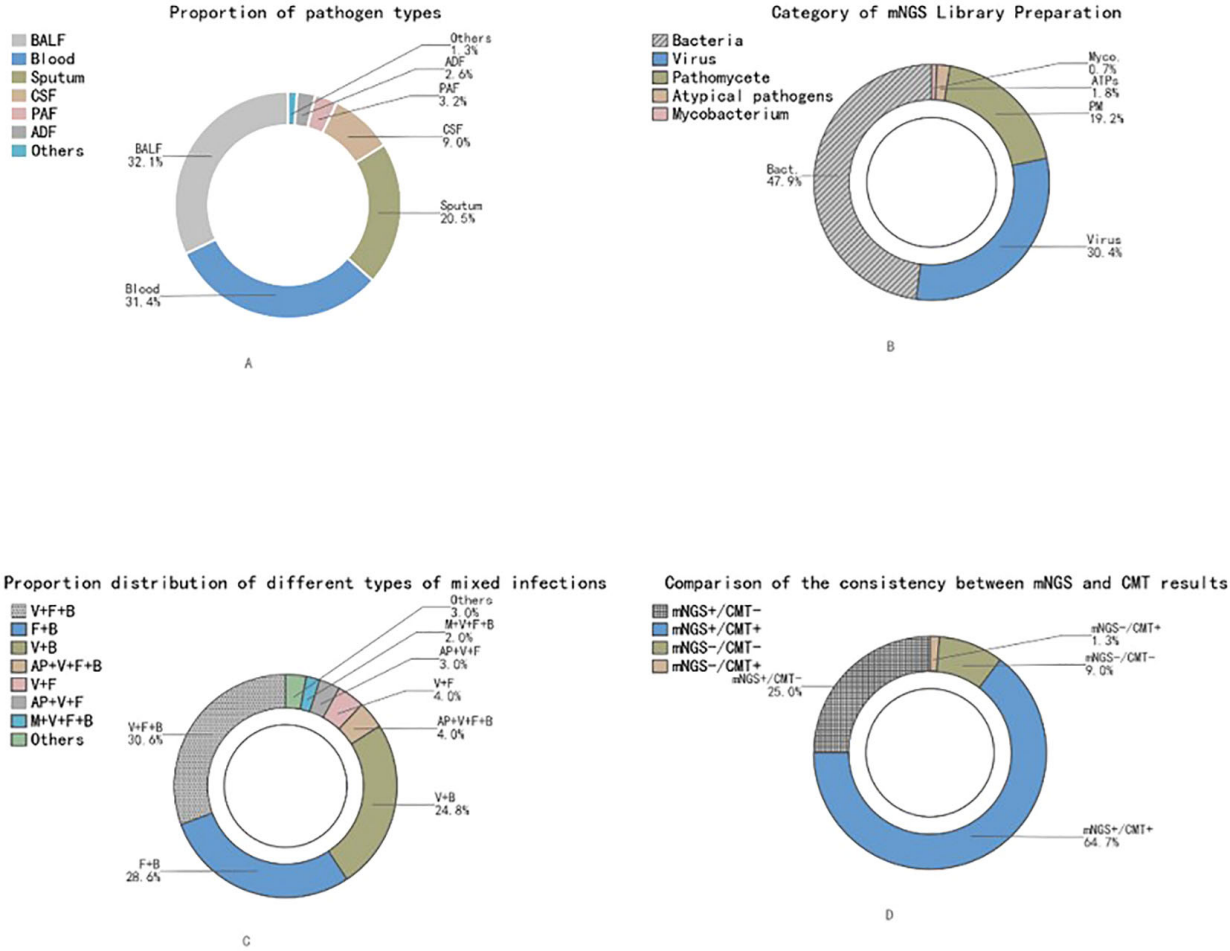
Analysis of pathogen detection characteristics based on mNGS technology. **(A)**: Distribution of sample source types for pathogens; **(B)** Proportion of different pathogen types in mNGS results; **(C)** Proportion of mixed infections with different pathogen combinations; **(D)** Consistency distribution between mNGS and CMT results. BALF, Bronchoalveolar lavage fluid; CSF, Cerebrospinal fluid; PAF, Pleural and ascitic fluid; ADF, Abscess drainage fluid; V, Virus; F, Fungus; B, Bacteria; AP, Atypical pathogen; M, Mycobacteria; CMT, Conventional microbiological testing).

The types of mNGS samples submitted aligned with clinical practice needs, primarily bronchoalveolar lavage fluid (BALF) with 50 cases (32.1%), followed by blood 49 cases (31.4%), sputum 32 cases (20.5%), cerebrospinal fluid 14 cases (9.0%), pleural/ascitic fluid 5 cases (3.2%, pleural fluid 3 cases, ascitic fluid 2 cases), abscess drainage fluid 4 cases (2.6%, liver abscess 2 cases, neck abscess 1 case, eye puncture fluid 1 case), and other specimens (e.g., midstream urine, stool) 2 cases (1.3%) (**Figure 1A**). This indicates that the study focused on ICU-infected patients in key sites such as the lower respiratory tract, bloodstream, and central nervous system.

mNGS detected a total of 447 pathogen strains, constructing an extremely complex microbial spectrum. **Figure 1B** shows a circular distribution chart of all detected pathogens, with bacteria accounting for the highest proportion (47.9%), followed by viruses (30.4%), fungi (19.2%), atypical pathogens (1.8%), and mycobacteria (0.7%). The most common bacteria, top five, were *Klebsiella pneumoniae* (14.5%), *Streptococcus pneumoniae* (14.0%), *Acinetobacter baumannii* (11.2%), *Corynebacterium striatum* (8.4%), and *Enterococcus faecium* (8.4%).

The most common fungi were *Candida albicans* (43.0%), *Candida glabrata* (18.6%), *Candida parapsilosis* (10.5%), *Candida tropicalis* (10.5%), and *Aspergillus fumigatus* (9.3%). The most common viruses were Epstein-Barr virus (EBV) (36.0%), cytomegalovirus (CMV) (19.1%), human herpesvirus type 1 (HHV) (19.1%), and human herpesvirus type 7 (5.9%). It is crucial to note that the detection of latent herpesviruses (like EBV, CMV) by mNGS does not automatically indicate active, causative disease, as it could represent asymptomatic reactivation, particularly in critically ill patients. In our study, clinical adjudication (see Methods 2.2.4) was applied to interpret the relevance of such findings in the context of each patient’s overall presentation.

A powerful capability of mNGS is revealing the full picture of mixed infections. This study diagnosed 101 cases of mixed microbial nucleic acid presence, accounting for 72.1% of total positive cases, far higher than the proportion of mixed microbial nucleic acid presence diagnosed by CMT positivity (34 cases, 33.0%). The most common types of mixed microbial nucleic acid presence were virus+fungus+bacteria (31 cases, 30.6%), fungus+bacteria (29 cases, 28.6%), and virus+bacteria (25 cases, 24.8%) combinations (**Figure 1C**). For example, one case of severe pneumonia simultaneously detected *Enterococcus faecalis*, *Aspergillus fumigatus*, human herpesvirus type 1, and SARS-CoV-2; another case of severe pneumonia in BALF simultaneously detected *Klebsiella aerogenes*, *Streptococcus pneumoniae*, *Acinetobacter baumannii*, *Mycobacterium tuberculosis* complex, and EBV. This high rate underscores the complexity of the ICU microbiome but also highlights the critical need for expert interpretation of mNGS data to avoid overdiagnosis of polymicrobial infections.

Further analysis of the consistency between mNGS and CMT results showed: 62.2% of patients were positive by both CMT and mNGS, 9.0% were negative by both. More importantly, among 53 CMT-negative samples, mNGS detected potential pathogenic microorganisms in 73.6% (39/53) of the samples, while among mNGS-negative samples, only 12.5% (2/16) were CMT-positive (**Figure 1D**). Among 103 CMT-positive cases, mNGS results for 51.5% (53/103) of the pathogens were consistent with conventional detection results at the genus or species level (Kappa = 0.225, P = 0.002).

**Figure 2:**
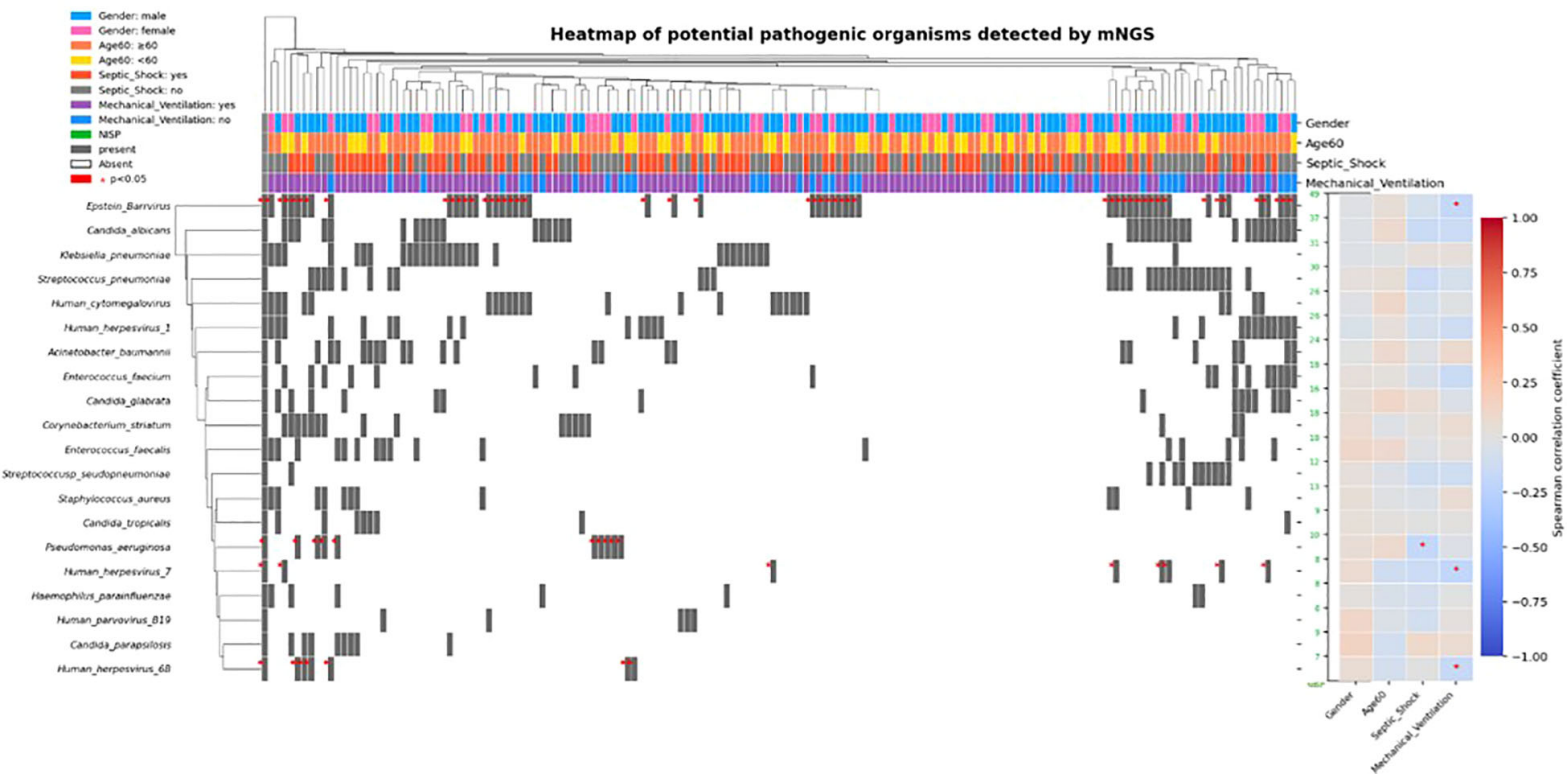
Heatmap of pathogen-phenotype-association strength (NISP: number of investigable sample pairs).

**Figure 2** integrates pathogen presence patterns, clinical phenotypes (sex, age, septic shock, mechanical ventilation), and correlation analysis, showing the top 20 potential pathogenic microorganisms detected by mNGS.

### 3.3 Comparison of diagnostic performance between mNGS and conventional testing methods

**Figure 3:**
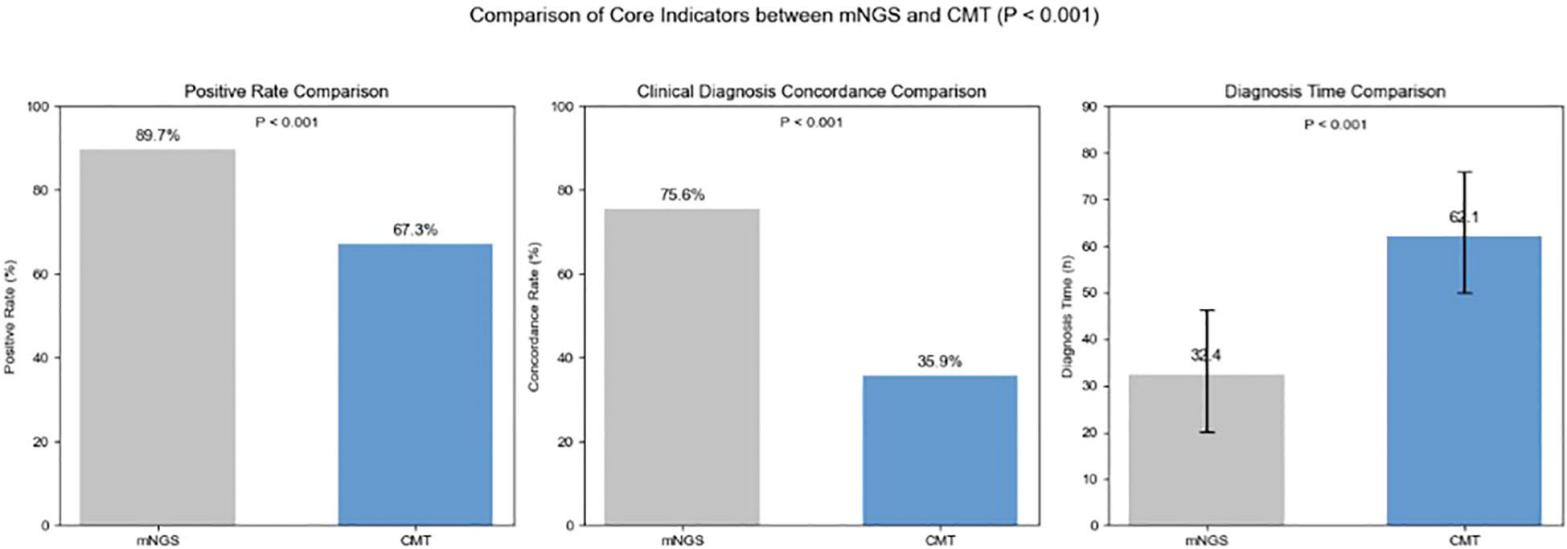
Comparison of core indicators between mNGS and traditional detection methods (CMT, conventional microbiological testing).

mNGS demonstrated overwhelming diagnostic advantages. As shown in **Figure 3A**, the total positive detection rate of mNGS was 89.7% (140/156), significantly higher than that of CMT at 67.3% (105/156), with a highly statistically significant difference (χ² = 24.905, P < 0.001). Using comprehensive clinical diagnosis as the gold standard, the overall concordance rate of mNGS was 75.6% (118/156), also significantly higher than that of CMT at 35.9% (56/156) (χ² = 49.947, P < 0.001) (**Figure 3B**). Apart from detection rate, diagnostic speed is another key factor affecting the prognosis of infectious diseases. We statistically analyzed the turnaround time (TAT) of mNGS and CMT. The average TAT for mNGS was 32.4 ± 12.2 hours (from sample submission to report issuance), while the average TAT for CMT and susceptibility testing was 62.4 ± 13.9 hours. The difference between the two was highly statistically significant (t = 20.187, P < 0.001) (**Figure 3C**). The TAT for mNGS was consistent across sample types and pathogen types (bacterial, viral, fungal), as the same wet-lab and bioinformatics pipeline was applied universally.

### 3.4 Analysis of resistance genes and virulence factors

#### 3.4.1 Resistance gene spectrum and its clinical correlation

**Figure 4:**
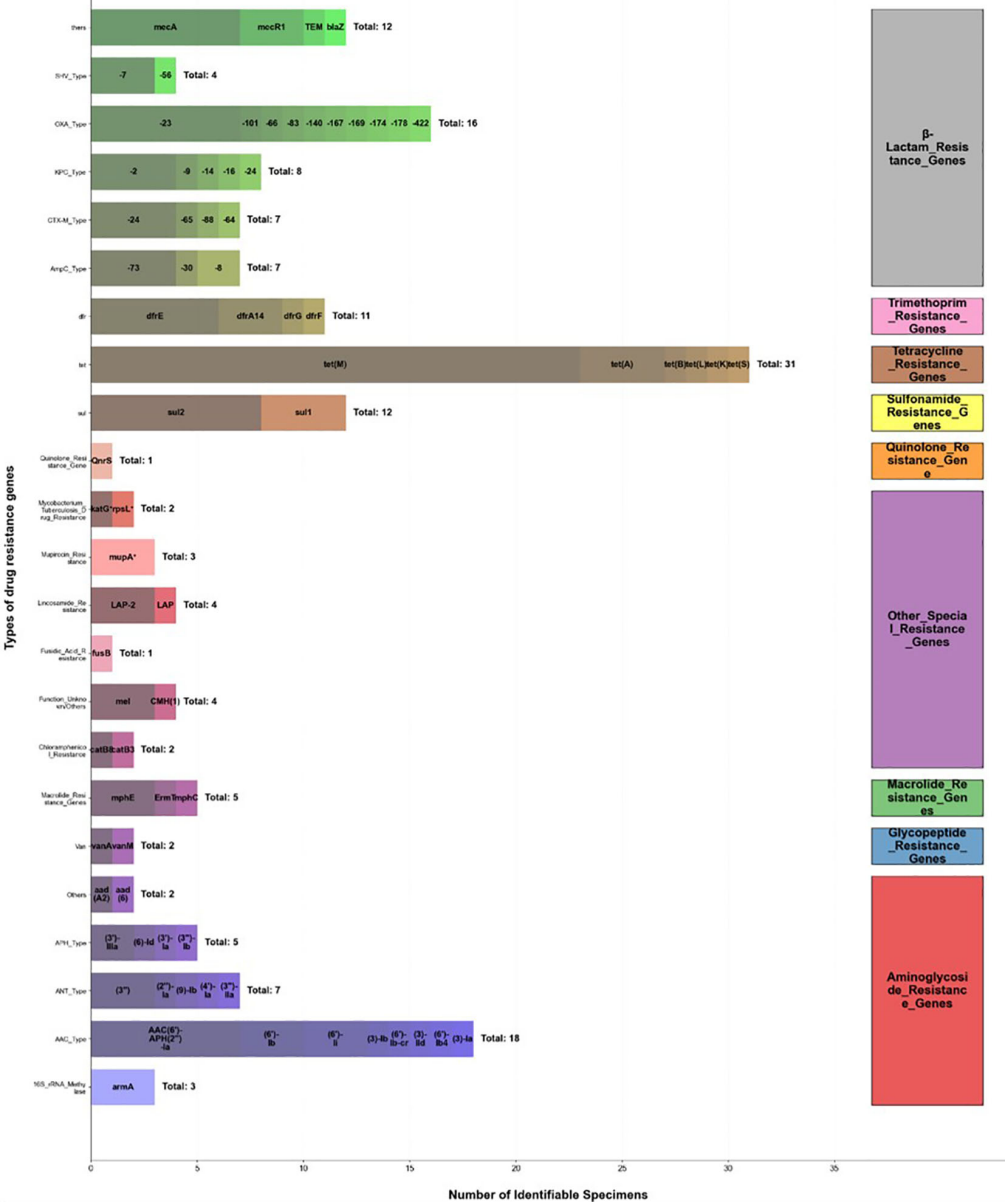
Distribution of resistance gene types detected by mNGS.

Deep mining of antibiotic resistance genes (ARGs) in mNGS data revealed a profound resistance background in the pathogen population. Among 102 patients determined to have bacterial infections, at least one clinically significant resistance gene was detected in the samples of 50 patients (49.0%), with a total of 169 specific ARGs detected (**Figure 4**). Among them, β-lactam resistance genes constituted the core of resistance (54 occurrences, 32.0%), with a complex internal structure: extended-spectrum β-lactamase (ESBL) genes (e.g., CTX-M-24, CTX-M-65, CTX-M-88, CTX-M-64, SHV-12, SHV-7) were detected 11 times; carbapenemase genes (e.g., KPC type, OXA type) were detected 24 times, including OXA-23 detected 7 times; AmpC enzyme genes (e.g., ADC-73, ADC-30, PDC-8) were detected 7 times. Other high-frequency resistance gene categories included tetracyclines 31 times (18.3%) and aminoglycosides 37 times (21.9%). This genotypic information showed a high degree of association with phenotypic susceptibility results. Among the 50 patients with detected resistance genes, in 36 cases (72.0%) the subsequent conventional susceptibility test results were consistent with the resistance gene predictions. We further analyzed the predictive value of carbapenemase genes: among 11 cases where KPC or OXA genes were detected, subsequent susceptibility testing confirmed resistance to meropenem or imipenem in 9 cases (81.8%).

#### 3.4.2 Virulence factor spectrum and its linkage analysis with host response

mNGS enables us to move beyond species identification to the molecular level of pathogen pathogenesis. In addition to resistance genes, mNGS can also reveal the virulence potential of pathogens. We constructed virulence factor profiles for major pathogens. For example, in *Streptococcus pneumoniae*, virulence genes related to adhesion and colonization, such as choline-binding proteins (PspC/CbpA), and *iga* which helps bacteria evade host mucosal immune defense were detected; in *Staphylococcus aureus*, *clfB*, encoding clumping factor B involved in bacterial colonization, was detected; in *Klebsiella pneumoniae*, virulence genes associated with hypermucoviscous phenotype, such as capsule synthesis (*rmpA*) and siderophores (*iroB*, *iutA*), were detected. Compared to *K. pneumoniae* infections lacking these virulence factors, infections with present virulence factors had higher PCT levels (13.0 [0.1, 28.0] ng/mL vs. 1.8 [0.4, 21.5] ng/mL) and higher mortality (20% vs. 16%), although without significant statistical difference. This provides a molecular-level explanation for understanding infection severity and pathogenesis.

### 3.5 Correlation between mNGS results and degree of inflammatory response/immune status

**Figure 5:**
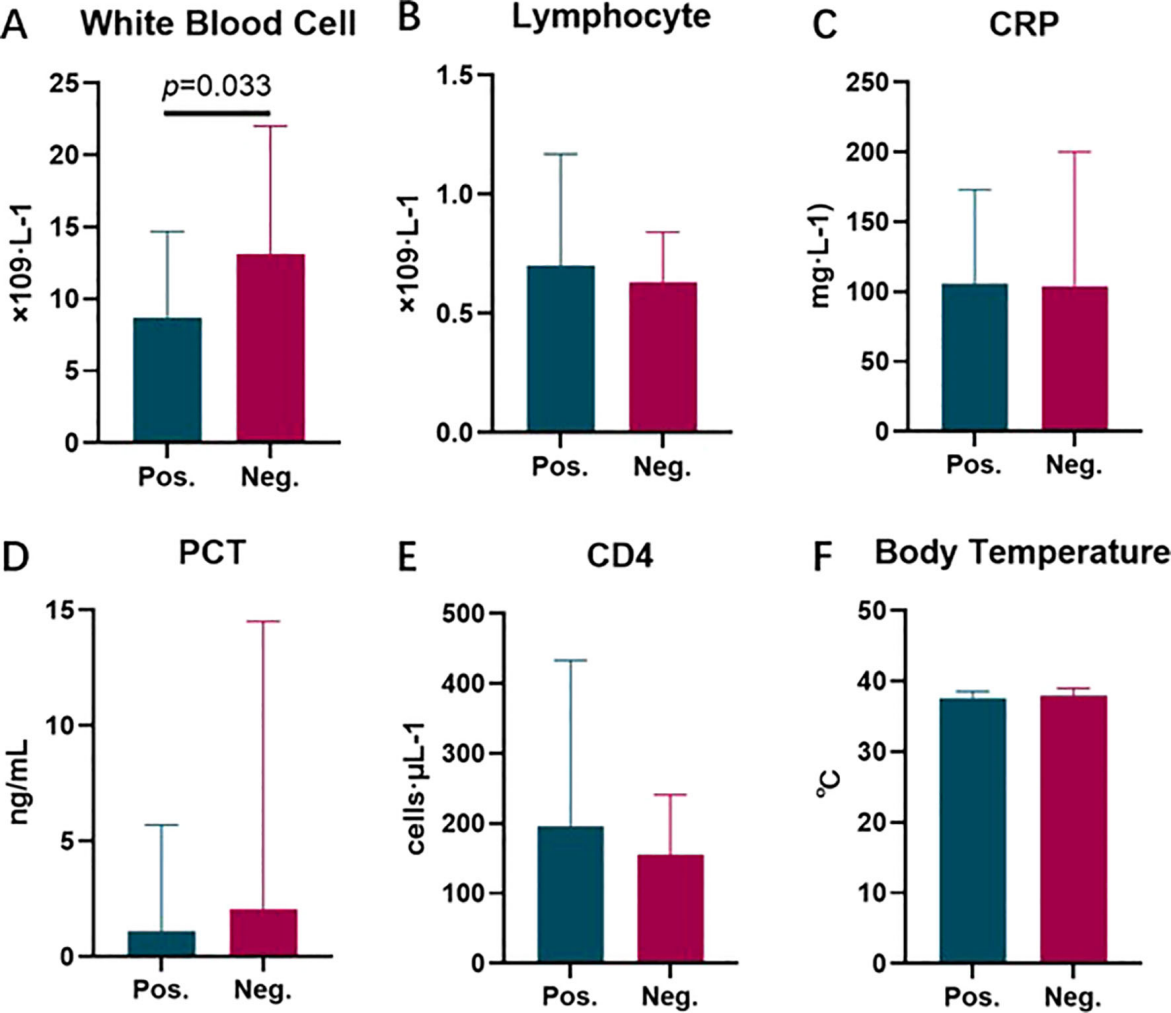
Comparison of Inflammatory Response Levels and Immune Status Between mNGS-Positive and Negative Groups. **(A)** Comparison of white blood cell count between groups; **(B)** Comparison of lymphocyte count between groups; **(C)** Comparison of CRP levels between groups; **(D)** Comparison of PCT levels between groups; **(E)** Comparison of CD4 count between groups; **(F)** Comparison of highest body temperature within 72 hours post-onset between groups.).

We compared the differences in multiple clinical indicators between the mNGS-positive and negative groups. The specific results are as follows: The WBC level in the mNGS-positive group was 8.71 [6.43, 14.71] × 10^9^/L, lower than that in the negative group at 13.13 [8.45, 22.00] × 10^9^/L, with a statistically significant difference between the two groups (p=0.033) (**Figure 5A**). The lymphocyte level in the positive group was 0.70 [0.45, 1.17] × 10^9^/L, higher than that in the negative group at 0.63 [0.43, 0.84] × 10^9^/L (**Figure 5B**). The CRP level in the positive group was 106 [48, 173] mg/L, while in the negative group it was 104 [58, 200] mg/L (**Figure 5C**). The PCT level in the positive group was 1.1 [0.3, 5.7] ng/mL, and in the negative group it was 2.1 [0.5, 14.5] ng/mL (**Figure 5D**). The CD4 level in the positive group was 196 [144, 433] cells/μL, and in the negative group it was 155 [113, 241] cells/μL (**Figure 5E**). The highest body temperature within 72 hours post-onset in the positive group was 37.6 (36.8, 38.6) °C, and in the negative group it was 37.9 (37.1, 39.0) °C (**Figure 5F**). The differences in lymphocytes, CRP, PCT, CD4, and temperature between the two groups were not statistically significant (p > 0.05).

### 3.6 Guiding value of mNGS results for clinical diagnosis and treatment

The rapid and accurate results of mNGS provided decisive basis for adjusting anti-infection strategies. Among the 156 patients, a total of 74 patients (47.4%) had their anti-infection regimens adjusted based on mNGS results. Adjustment strategies mainly included: De-escalation therapy (28 cases, 17.9%): switching from broad-spectrum antibiotics to narrower-spectrum, more targeted antibiotics when mNGS did not detect resistant bacteria or only detected susceptible bacteria; Escalation/targeted therapy (31 cases, 19.9%): timely addition or switching to targeted drugs when mNGS detected resistant bacteria (e.g., MRSA, CRKP) missed by conventional culture or special pathogens (e.g., *Aspergillus*, *Chlamydia*); Antiviral therapy (10 cases, 6.4%): adding antiviral drugs like ganciclovir when mNGS indicated a virus was the main causative pathogen or an important synergistic factor; Discontinuation of unnecessary antibiotics (5 cases, 3.2%): assisting clinical decision-making to stop antibiotics when mNGS results were negative or only detected clinically insignificant colonizing bacteria.

## 4 Discussion

Through a relatively large clinical cohort, this study systematically validated the comprehensive value of mNGS in the diagnosis of critical infections. Its core contribution lies in elevating the output of mNGS from a simple “list of pathogens” to an “integrated report” that combines pathogen identity (Species), resistance potential (Resistance), virulence attributes (Virulence), and links them with host response (Host Response). This provides a powerful tool for precision medicine practice in infectious diseases.

First, our data on diagnostic timeliness (TAT) provides one of the most convincing pieces of evidence for the clinical advantage of mNGS. Obtaining an etiological diagnosis 30 hours earlier means empirical therapy can be converted to targeted therapy sooner. This not only may improve patient prognosis but also directly aligns with the core principles of antimicrobial stewardship (AMS), holding significant public health importance for curbing the spread of microbial resistance (Graf et al., 2016). Furthermore, consistent with previous studies (Chen et al., 2021; Yang et al., 2022), we confirmed that the pathogen detection rate of mNGS is significantly higher than that of CMT, primarily due to its independence from microbial growth *in vitro* and high-throughput detection capability. This study also highlights the great potential of mNGS in revealing the true picture of “mixed infections.” Nearly three-quarters of mNGS-positive cases involved mixed infection, a proportion far exceeding that of CMT. In addition, mNGS is not affected by the use of antibiotics, which may lead to the low consistency between mNGS and CMT results. More importantly, the concordance rate between mNGS and clinical diagnosis reached 75.6%, also far surpassing CMT. These provide crucial clues for pathogen diagnosis, holding extremely high etiological diagnostic value.

Our analysis of resistance genes (ARGs) goes beyond simple “presence or absence” descriptions to a quantitative assessment of their clinical predictive value. We observed that the positive predictive value (PPV) of mNGS for carbapenemase genes was as high as 81.8%, consistent with conclusions from several recent studies (Charalampous et al., 2019; Liu et al., 2022; Tian et al., 2025). This suggests that “preemptive therapy” based on mNGS resistance gene reports before susceptibility results are available is highly reliable, especially in CMT-negative cases. Additionally, our exploration of virulence factors (VFs) pushes the understanding of infection pathology to the molecular level. We found that high-virulence genes (e.g., *rmpA/A2*, *iucABCD-iutA* in hvKP) are not just molecular tags of bacteria but also drivers of intense host immune-inflammatory responses (Russo and Marr, 2019; Sheng et al., 2025). The significantly elevated PCT levels in patients are a macroscopic manifestation of this intense “pathogen-host” interaction. This suggests that future mNGS reports may not only guide antibiotic selection but also provide new molecular markers for assessing disease severity and predicting complication risks, even laying the groundwork for developing novel anti-virulence therapies targeting specific virulence factors (Assoni et al., 2024).

Interestingly, while it was previously believed that mNGS had higher positivity rates in individuals with strong inflammatory responses and low immune levels (Li et al., 2022; Gao et al., 2025; Zheng et al., 2025), our study found that mNGS has diagnostic value across varying degrees of host inflammation and immune status. This indicates its broad applicability, not limited to hosts with extremely low immunity.

Of course, as a retrospective analysis, this study has inherent limitations. First, there may be selection bias, e.g., patients with more complex conditions or those negative by traditional methods are more likely to be sent for mNGS testing. Second, standardization of mNGS testing and interpretation of results, especially distinguishing low-sequence-count pathogens from colonizers, remain challenges for the industry.Our reported high rate of mixed nucleic acid detection should be interpreted with this key limitation in mind. Third, the association of detected ARGs/VFs with specific bacterial species is inferential when multiple bacteria are present, and functional validation of these genes was not always possible. Future prospective, multicenter randomized controlled trials are needed to ultimately confirm whether diagnostic and therapeutic strategies based on the “integrated report” of mNGS can unequivocally reduce mortality in critically ill infected patients and optimize healthcare resource utilization.

## 5 Conclusion

mNGS represents a paradigm shift in infectious disease diagnostics. By concurrently illuminating the pathogen’s identity, its resistance and virulence arsenal, and the host’s response, it delivers a multidimensional and integrative perspective on infection. This comprehensive approach is foundational for advancing precision antimicrobial therapy and strengthening the fight against AMR in the management of ICU-infected patients.

## Data Availability

The raw data supporting the conclusions of this article will be made available by the authors, without undue reservation.
