# Supplementary figures and images for: Integrative analysis of pathogen detection, antimicrobial resistance, virulence, and host response in severe infections using metagenomic next-generation sequencing
Source: Front Cell Infect Microbiol. 2026 Apr 10;16:1786413. doi: 10.3389/fcimb.2026.1786413 (PMC13105863; doi:10.3389/fcimb.2026.1786413)

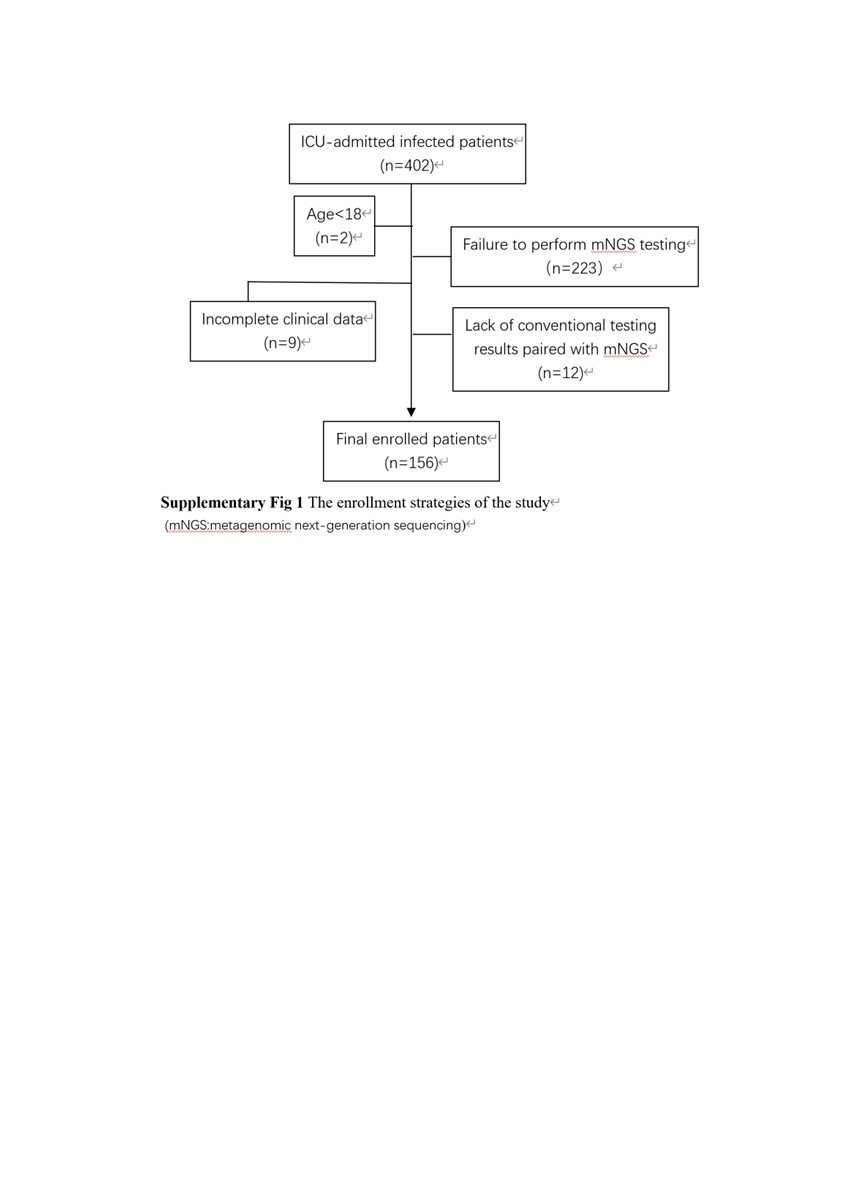

Supplement: Supplementary file 1 [file Image1.jpeg]
