# Supplementary material for: Integrative analysis of pathogen detection, antimicrobial resistance, virulence, and host response in severe infections using metagenomic next-generation sequencing
Source: Front Cell Infect Microbiol. 2026 Apr 10;16:1786413. doi: 10.3389/fcimb.2026.1786413 (PMC13105863; doi:10.3389/fcimb.2026.1786413)
Supplement: Supplementary file 2 [file Table1.docx]

**Metagenomic Next-Generation Sequencing (mNGS) and Bioinformatics Analysis**

**2.2.2.1 Sample Processing, Nucleic Acid Extraction, and Host Depletion**Patient samples, including bronchoalveolar lavage fluid (BALF), blood, cerebrospinal fluid (CSF), sputum, and others, were collected under sterile conditions and processed within 2 hours of collection. Total nucleic acid (DNA and RNA) was co-extracted from 300 μL of each sample using the TIANamp Micro DNA Kit (DP316, Tiangen, China) for DNA and the QIAamp Viral RNA Mini Kit (52906, Qiagen, Germany) for RNA, according to the manufacturer’s protocols. To improve microbial signal and reduce human background, host depletion was performed on DNA extracts using the NEBNext® Microbiome DNA Enrichment Kit (E2612S, NEB, USA) targeting methylated human DNA, following the standard protocol.

**2.2.2.2 Library Preparation and Sequencing**Extracted DNA and RNA were processed separately for library construction. For RNA libraries, ribosomal RNA (rRNA) was depleted using the Ribo-Zero™ Human/Mouse/Rat Kit (Illumina, USA), followed by reverse transcription using random hexamers and the SuperScript™ IV First-Strand Synthesis System (Thermo Fisher, USA). DNA and cDNA were then fragmented to an average size of 200–300 bp using a Covaris M220 focused-ultrasonicator (Covaris, USA). Sequencing libraries were prepared using the NEBNext® Ultra™ II DNA Library Prep Kit for Illumina (NEB, USA) with dual indexing adapters. Libraries were quantified using a Qubit 4.0 Fluorometer (Thermo Fisher, USA) and pooled in equimolar ratios. Sequencing was performed on an Illumina NextSeq 550 platform using a 2×150 bp paired-end configuration. The target sequencing depth was set to a minimum of 20 million raw reads per sample to ensure adequate coverage for low-abundance pathogens.

**2.2.2.3 Bioinformatic Pipeline for Taxonomic Identification**Raw sequencing data were subjected to quality control using FastQC (v0.11.9) and Trimmomatic (v0.39) to remove low-quality bases (quality score < Q20), adapter sequences, and reads shorter than 50 bp. High-quality reads were aligned to the human reference genome (GRCh37/hg19) using Bowtie2 (v2.4.4) to remove host-derived sequences. Non-human reads were retained for subsequent microbial analysis.

Taxonomic classification was performed using a two-step approach: (1) Reads were classified using Kraken2 (v2.1.2) against a custom microbial database containing bacterial, viral, fungal, and parasitic genomes from NCBI RefSeq; (2) To improve specificity, reads assigned to potential pathogens were further mapped to their respective reference genomes using BWA-MEM (v0.7.17) to calculate genome coverage and confirm identification. A pathogen was considered detected if it met all of the following criteria:

1. Read count threshold: ≥10 uniquely mapped reads for bacteria/fungi, ≥5 for viruses/atypical pathogens.
2. Relative abundance threshold: Reads per million (RPM) ≥10 for bacteria/fungi, RPM ≥5 for viruses/atypical pathogens.

(3) Negative control subtraction: Any organism present in the negative-control sample at RPM ≥1 was considered a potential contaminant and excluded from clinical reports unless its abundance in the patient sample was at least 10-fold higher.

**2.2.2.4 Resistance Gene and Virulence Factor Analysis**Non-human reads were aligned against the Comprehensive Antibiotic Resistance Database (CARD, version 3.2.5) and the Virulence Factor Database (VFDB, 2022 release) using BLASTn (v2.12.0+) with an E-value cutoff of 1×10⁻⁵. A resistance gene (ARG) or virulence factor (VF) was considered “present” if the alignment met the following criteria: ≥95% nucleotide identity over ≥90% of the reference gene length. Read counts for each ARG/VF were normalized to RPM. For samples with a clearly dominant bacterial pathogen (constituting >50% of bacterial reads), detected ARGs and VFs were tentatively associated with that pathogen.

**2.2.2.5 Quality Control and Validation**Each sequencing batch included:

(1) Negative controls: Sterile nuclease-free water was processed alongside clinical samples through extraction and library preparation to monitor background contamination.

(2) Positive controls: The ZymoBIOMICS Microbial Community Standard (D6300, Zymo Research, USA) was used to validate sensitivity, specificity, and limit of detection of the workflow.
Data from negative controls were used for background subtraction, and only organisms/genes not detected in controls (or significantly higher in clinical samples) were reported.

**2.2.2.6 Data Availability and Reproducibility**All custom scripts and pipelines used for bioinformatic analysis are available upon reasonable request. The version numbers of all software and databases are documented above to ensure reproducibility.
